# The Effect of Attribute Alignability on Product Purchase: The Moderating Role of Product Familiarity and Self-Construal

**DOI:** 10.3389/fpsyg.2021.636922

**Published:** 2021-03-31

**Authors:** Yong Zhang, Yuwen Wen, Min Hou

**Affiliations:** School of Management, Jinan University, Guangzhou, China

**Keywords:** attribute alignability, product familiarity, self-construal, product purchase, perceived diagnosticity, differentiation

## Abstract

Previous studies on the Structural Alignment Model suggest that people compare the alignable attributes and nonalignable attributes during the decision-making process and preference formation process. Alignable attributes are easier to process and more effective in clue extracting. Thus, it is believed that people rely more on alignable than nonalignable attributes when comparing alternatives. This article supposes that consumers’ product experience and personal characteristics also play a significant role in regulating consumers’ reliance on attribute alignability. The authors conducted three experiments to examine the moderating role of consumers’ product familiarity and self-construal in the impact of attribute alignability on consumer product purchase. The results show the following: (1) When making a purchase decision, consumers with a high level of product familiarity will rely more on nonalignable attributes, while those with a low level of product familiarity will rely more on alignable attributes. (2) The difference in consumer dependency on attribute alignability is driven by their perceived diagnosticity of attributes. (3) The dependency of consumers with different levels of familiarity on attribute alignability will be further influenced by consumers’ self-construal. Individuals with interdependent self-construal rely more on alignable attributes when unfamiliar with the product, while relying more on nonalignable attributes when familiar with the product. Individuals with independent self-construal, however, rely more on nonalignable attributes regardless of the degree of product familiarity. The conclusions of this paper can be used as references for enterprises to establish product positioning and communication strategies.

## Introduction

To stay competitive in today’s rapid-changing marketplace, many enterprises attempt to make their products or brands outstanding by establishing differentiations. Thus, they manage to construct a constantly competitive advantage that is difficult for competitors to replicate or imitate. For example, when a mobile phone brand releases new products, the product attributes will be different from those of competitors. Some of the differentiations can be compared. For example, the weight of a Huawei P40 Pro is 209 g, while that of an iPhone11 is 194 g. Some of the differentiations, however, are unique. For example, the Huawei P40 Pro+ supports 5G network, while the iPhone11 supports Deep Fusion (a new computational photography process specifically on the iPhone 11 line).

For enterprises that strive to better display their competitive advantages, should they differentiate their products on alignable attributes (comparable attributes) or nonalignable attributes (incomparable unique attributes) in the product design process? When positioning products and formulating communication strategies, can they better attract consumers by highlighting the advantages of alignable attributes or nonalignable attributes? Do consumers choose new products based on the attributes that can be directly compared, or the unique attributes that are difficult to compare? Would consumers with different characteristics have different preferences?

In recent years, Scholars have discussed the influence of attribute alignability on consumers’ choices and judgments based on different attributes. Early studies suggested that alignable attributes (vs. nonalignable attributes) have a greater weight in consumers’ purchase decision-making (Markman and Gentner, 1997; Zhang and Markman, 1998; Zhang and Fitzsimons, 1999). Later, however, some scholars believed that personal or situational factors, including the need for cognitive closure (Zhang et al., 2002), the degree of intervention (Zhang and Markman, 2001), the level of temporal construal (Malkoc et al., 2005), the evaluation mode (Sun, 2011), and uncertainty (Sun et al., 2012), can prompt consumers to rely more on nonalignable attributes. Nevertheless, few literatures discuss the important effect of product familiarity on consumers’ dependency on attribute alignability. Due to the widespread use of internet and the increase in advertising channels, different consumers show great differences in different products. Product familiarity is a simple, effective, and extensive way of consumer segmentation. Consumers with different level of product familiarity show great differences in mindset, preference formation, and behavior tendency. Furthermore, Taylor-West et al. (2020) suggest that marketing departments should review their data capture methods to collect more basic consumer information on their level of familiarity. Therefore, it is meaningful to further explore the moderating role of product familiarity in consumers’ dependency on attribute alignability.

Additionally, influenced by cultural and situational factors, how consumers view themselves also has an important impact on new product adoption. This makes it necessary to enrich the research on the moderating effect of consumers’ individual characteristics. It is essential to further explore the consumer cognition in decision-making process.

This study introduces product familiarity and self-construal into the research model and further discusses the moderating role of product familiarity and self-construal in the influence of attribute alignability on consumer product purchase. Specifically, this study will discuss the following questions: (1) Whether product familiarity will affect consumers’ dependency on attribute alignability in purchase decision-making. (2) Whether consumers with different self-construal and product familiarity rely differently on attribute alignability. The discussion of these questions is helpful in developing a more profound understanding of consumers’ decision-making process, providing relevant marketing strategies and suggestions for enterprises.

## Theory and Hypotheses

### The Structural Alignment Model

Comparing alternatives plays an important role in consumers’ decision-making process. Contrast Model in psychology was an early model. It held that similarity judgments are the result of comparing common and distinctive features (Tversky, 1977). Later, scholars established the Structural Alignment Model to explain the consumer’s comparative decision-making process by extending the Contrast Model (Markman and Gentner, 1993; Gentner and Markman, 1997). The Structural Alignment Model further categorized the attribute generated in the comparison process. Specifically, consumers would compare the similarities and differences of competitive brands, and the differences can be divided into alignable attributes and nonalignable attributes. Alignable attributes are the attributes that all alternative brands have in common, but might be different in magnitude. A nonalignable attribute is owned by only one brand, which can be a unique attribute owned by this product or an attribute not involved by other alternatives.

Early studies have shown that people rely more on alignable attributes than nonalignable attributes when making comparisons and decisions (Markman and Gentner, 1993; Medin et al., 1995; Nam et al., 2012). Compared with nonalignable attributes, alignable attributes are easier to remember (Zhang and Markman, 1998), more effective in clue extracting (Markman and Gentner, 1997), and more often used to describe the differences between two options (Gentner and Markman, 1994). However, it is also found that in the decision-making process, people pay more attention to nonalignable attributes than to alignable attributes. When influenced by some personal factors or situational factors, such as motivation (Zhang and Markman, 2001), evaluation mode (joint evaluation vs. separate evaluation; Sun, 2011), expertise (Sun et al., 2012), and regulatory orientation (promotion orientation vs. prevention orientation; Sun et al., 2019), the influence of nonalignable attributes on consumers’ decision-making is more prominent, which will make consumers rely more on nonalignable attributes. Therefore, consumers’ dependence on alignable or nonalignable attributes is not monotonous.

Product familiarity is a prominent factor that has great impact on consumers’ mindset, preference formation, and behavior tendency. Based on the Structural Alignment Model, we further explore the impact of product familiarity on attribute alignability dependency. Although Nam et al. (2012) have examined that consumer expertise has a great impact on consumers’ dependency on attribute alignability, we considered that consumer expertise and product familiarity are two different constructs, conceptually and practically. Alba and Hutchinson (1987) indicated that familiarity represents the early stages of learning, while expertise represents the later stages of learning. Gursoy and Chi (2008) supposed that familiarity represents subjective knowledge, while expertise represents objective knowledge. Familiarity, therefore, is described as the awareness or perception of a product/service (Kerstetter and Cho, 2004). Familiarity is a feeling-based perception, while expertise is a knowledge-based perception. For example, many people are familiar with cars, but only a few of people have expertise in them. This makes it meaningful to further explore the important role of product familiarity in consumers’ dependency on attribute alignability (Table 1).

**Table 1:** The development of the Structural Alignment Model.

Findings	Author(s), year
Proposing the Contrast Model. Objects are collections of features and similarity is described as a feature matching process. Similarity judgments are the result of comparing common and distinctive features.	Tversky, 1977
Proposing the Structural Alignment Model. It should be easier to find the differences between pairs of similar items than between pairs of dissimilar items.	Markman and Gentner, 1993; Gentner and Markman, 1994, 1997
There is an important correspondence between similarity processing and decision-making process.	Medin et al., 1995
Compared with nonalignable attributes, alignable attributes are easier to remember and more effective in clue extracting.	Markman and Gentner, 1997; Zhang and Markman, 1998
High motivation to process information enables consumers to increase their use of nonalignable differences in preference formation.	Zhang and Markman, 2001
The influence of alignability on evaluation is moderated by the need for cognitive closure, which influences preferences for easy comparison and less ambiguity.	Zhang et al., 2002
When influenced by evaluation mode, uncertainty, expertise, self-construal, and regulatory orientation, the influence of nonalignable attributes on consumers’ decision-making is more prominent.	Sun, 2011; Nam et al., 2012; Sun et al., 2012, 2019; Lee and Lee, 2016

### The Impact of Product Familiarity on Attribute Alignability Dependency

When evaluating the utility of a product, consumers need to have a certain degree of experience or prior knowledge in order to evaluate the functional attribute of the product, especially new products (Hsee, 1996). A customer’s prior experience with the product, that is, product familiarity, will affect consumers’ perception and evaluation of product attributes (Alba and Hutchinson, 1987). Once consumers get familiar with products, innovative and unique attributes can be more easily understood (Rogers, 2003). Furthermore, experienced consumers will pay more attention to new, interesting, and unique attributes of the product and think that the alignable attributes are consistent or redundant (Kardes and Kalyanaram, 1992). They believe that more unexpected surprises and value can be brought by the unique, nonalignable attributes of the product (Murshed et al., 2018). Sun et al. (2019) also stated that when evaluating alternative products, if relative ease of processing is the reason why consumers rely more on alignable attributes rather than nonalignable attributes, consumers will rely more on nonalignable attributes when they have the ability to overcome the difficulties of processing information. As for low-familiarity consumers, nonalignable attributes are considered to have higher inconsistencies (Zhou and Nakamoto, 2007) and higher degree of uncertainty (Heath and Tversky, 1991). When consumers make a decision, they will first recall the relatively simple and comparable differences that have been presented (Zhang and Markman, 1998), then make choices accordingly. Many advertisements also indicate that products with high public familiarity, such as mobile phones, tend to highlight their nonalignable attributes (e.g., the iPhone 11 highlights Deep Fusion, a unique photo taking function), while products with low public familiarity, such as cameras, tend to highlight their alignable attributes (e.g., the Canon EOS R5 emphasizes its 8 K resolution and 20 fps continuous shooting speed).

Therefore, we proposed the following hypothesis:

Hypothesis 1: When consumers have a high degree of product familiarity, they will rely on the nonalignable attribute to make purchase decisions. When consumers have a low degree of product familiarity, they will rely on alignable attributes to make purchase decisions.

### The Mediating Role of Perceived Diagnosticity

In a study on the Accessibility-Diagnosticity Model in memory choices, Lynch et al. (1988) pointed out that the information diagnostic is the degree to which decision makers believe that information itself can help them achieve their decision-making goals. Therefore, diagnosticity is decision makers’ subjective evaluation of the usefulness of information driven by decision goals. The more diagnostic the information is, the more it will be used by decision makers in decision-making and judgment.

Existing literature shows that alignable attributes have a greater impact on consumption decisions than nonalignable attributes (Markman and Gentner, 1993). Consumers often need to evaluate product attributes from their past consumption experience and other data or clues when making decisions. The less experience the consumers have, the less likely they are to believe their own inferences, meaning that they turn to rely more on the information clearly provided. Therefore, it is relatively easy to deal with alignable attributes for novices or consumers with less product experience, which makes alignable attributes more diagnostic than nonalignable attributes (Feldman and Lynch, 1988). However, with further research, more scholars believe that consumers rely more on nonalignable attributes under many conditions. For example, Zhang and Markman (2001) believe that as consumers’ involvement increases, consumers are more dependent on nonalignable attributes in product preferences. Nam et al. (2012) believe that experts (vs. novices) are more likely to be motivated by nonalignable attributes that need to consume cognitive resources for processing. Experts think that nonalignable attributes are more differentiated and diagnostic and therefore will be more likely to use nonalignable attributes in decision-making. In short, for consumers with a high degree of product familiarity, nonalignable attributes are more diagnostic. We believe that consumers with different product experience separately rely on alignable and nonalignable attributes, driven by their perception of diagnosticity of attributes.

Therefore, we proposed the following hypothesis:

Hypothesis 2: the interaction effect of attribute Alignability and product familiarity on product purchase is mediated by perceived diagnosticity.

### The Influence of Product Familiarity and Self-Construal on Attribute Alignability Dependency

Consumers’ dependency on alignable and nonalignable attributes is affected not only by their product experience but also by individual differences (Nam et al., 2012). Based on the cultural influence on the formation of individual self-systems, Markus and Kitayama (1991) proposed the concept of “self-construal.” They supposed that self-construal, people’s perception of themselves, differs according to the degree of connection between themselves and others in society. Individuals with independent self-construal consider themselves to be autonomous and separate from others, while those with interdependent self-construal consider themselves to be connected with others. Self-construal has also been extensively studied in the consumption domain, mainly relevance to brand association (Escalas and Bettman, 2005), product characteristics (Lee and Kacen, 2008; Ma et al., 2014), information processing (Heine et al., 1999; Lee et al., 2000; Aaker and Schmitt, 2001; Jain et al., 2007), advertising persuasion (Han and Shavitt, 1994), etc.

Different self-construal will induce differentiated consumption goals and have an impact on consumer’s psychology and decision-making. Independents pursue the difference between themselves and others and are more willing to take risks (Aaker and Schmitt, 2001). Interdependents attach importance to consistency and harmony with others, exhibit convergent effects in behavior, and are more willing to avoid risks.

When familiar with products, independents can be positively affected by unique products (Carpenter et al., 1994), making them rely more on nonalignable attributes. In addition, independents tend to have positive attitudes and dare take risk when making decisions even without familiarity with product (Hamilton and Biehal, 2005). In order to match products with their own distinctive characteristics, they will search more information on nonalignable attributes of products to reduce perceived uncertainty and improve the diagnosability of information and ultimately choose products with better nonalignable attributes.

When unfamiliar with products, due to the lack of product experience, interdependents will perceive greater uncertainty and risks when confronted with nonalignable attributes (Ma et al., 2014). For the purpose of avoiding risks, they will rely on alignable attributes to make purchase decisions. When familiar with products, the rich experience can reduce their perceived risks and uncertainty. This makes interdependents believe that they have the ability to process information of nonalignable attributes and prefer products with better nonalignable attributes that can meet their unique demand.

In view of the reasoning set out here, we proposed the following hypothesis:

Hypothesis 3a: Regardless of the consumer’s product familiarity, independents significantly rely more on nonalignable attributes than on alignable attributes.
Hypothesis 3b: Interdependents significantly rely more on alignable attributes when they have a low degree of product familiarity, while relying more on nonalignable attributes when they have a high degree of product familiarity.

## Materials and Methods

### Study Overview

**Figure 1:**
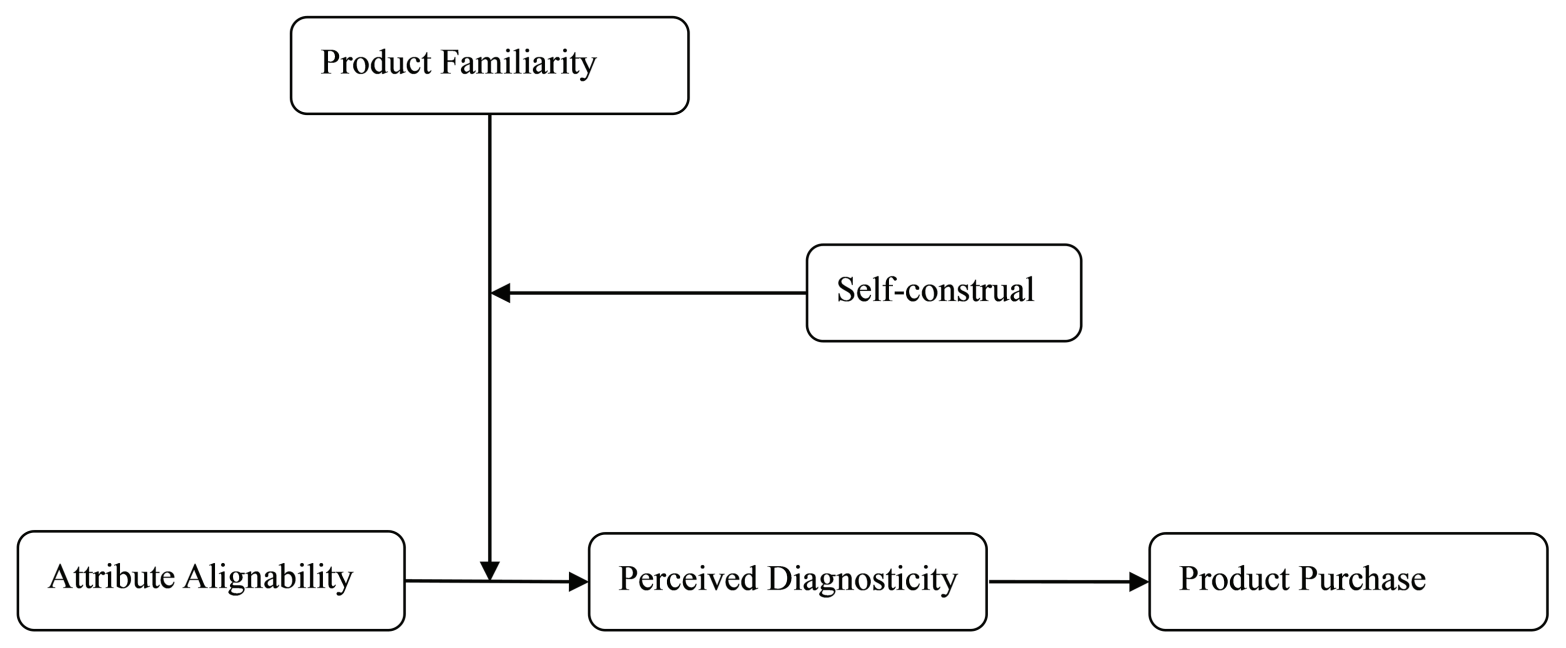
The research model.

In conclusion, the research model of this article is shown in Figure 1. We validated the above hypothesis through three experiments. The first study examines the influence of product familiarity on the dependency on attribute alignability and the mediating effect of perceived diagnosticity, that is, to test H1 and H2. Studies 2 and 3, respectively, examine the interaction effects of product familiarity and self-construal on the dependency on attribute alignability in the context of different product category and the same product category, that is, to test H3a and H3b.

### Study 1

#### Pretest and Stimuli

Most of the participants in the experiment are undergraduates. Taking their product knowledge into account, we chose mobile phones, with which the participants were very familiar, as the stimulus of the familiar product group, while choosing a portable monitoring player, a fictitious product, as the stimulus of the unfamiliar product group (Zhou and Nakamoto, 2007). In order to eliminate the influence of brand preference, we used two fictional brands, brand A (superior in alignable attributes) and brand B (superior in nonalignable attributes) for mobile phone and portable monitoring player, respectively. To be more specific, the alignable attributes of brand A are better than those of brand B, while the nonalignable attributes of brand B are better than those of brand A. Then, we conducted two pretests to ensure the validity of the experimental materials.

Thirty-six undergraduate students participated in the first pretest to filter the mobile phone’s attributes we used in the experiment. According to the interviews with several mobile phone users, we listed 35 attributes of the mobile phone in the survey and asked participants to rate the importance of each attribute when making purchase decision on a seven-point scale (1 = not important at all, and 7 = very important). Based on these results, we selected the 12 most important attributes (screen size, rear camera pixel, battery capacity, phone memory, resolution, recording function, compatible with multiple operators, CPU performance, front camera pixel, Wi-Fi function, autofocus, and sound quality) and randomly divided them into three blocks of four attributes each. To ensure that the importance of attributes in three blocks have no significant difference, we randomly recruited 30 students to rate the importance of three blocks in the second pretest. Importance ratings showed no significant difference across the three blocks of mobile phone attributes [M_1_ = 5.70, M_2_ = 5.71 vs. M_3_ = 5.70; *F*(2,87) = 0.013, *p* > 0.05].

#### Participants and Design

One hundred and sixty students (49.2% females, aged from 18 to 25 years) were recruited to participate in this experiment in exchange for monetary compensation. Study 1 employed a 2 (brand: brand A with superior alignable attributes vs. brand B with superior nonalignable attributes) × 2 (product familiarity: familiar vs. unfamiliar) mixed design, with the first factor as a within-subjects factor and the second factor as a between-subjects factor. The three blocks of attributes of both brands were assigned by Latin square design, to ensure that participants’ preference for brand A or B was driven only by the difference between alignable and nonalignable attributes. Participants were randomly assigned to either the familiar or unfamiliar condition. The return of questionnaires with valid responses was 82.5%.

##### Procedure

Firstly, participants were asked to rate their familiarity with the product on two items using a seven-point scale (1 = not familiar at all, 7 = very familiar; Coupey et al., 1998). Then, we presented the basic information of the two brands through PowerPoint and explained the concepts of alignable and nonalignable attributes to the participants. Next, the participants were asked to complete the rest of the questionnaire, which consists of three parts. The first part was the detailed description of the product information, presenting the alignable and nonalignable attributes of the two brands in a matrix separately. Participants were asked to read the information of the two brands carefully and complete several questions about their understanding of the reading material. In the second part, participants were asked to evaluate the attractiveness of each attribute, the purchase intention of two brands (Sweeney et al., 1999), the brand evaluation (Kardes and Kalyanaram, 1992), and the perception of diagnosticity (Pham and Avnet, 2004). In the third part, participants filled in their demographics. The entire experiment process was completed in approximately 30 min.

#### Results

##### Manipulation Check

The manipulation check confirmed the effectiveness of all our manipulation. For those in the familiar and unfamiliar conditions, we averaged the two familiarity items to create a familiarity index. The results revealed that the participants in the familiar condition who finished the survey regarding the mobile phone were indeed more familiar with the product than those in unfamiliar condition who finished the survey regarding the portable monitoring player (M_phone_ = 6.00, M_monitoring player_ = 2.24, *p* < 0.001). Moreover, our construction of the brands based on attribute attractiveness is appropriate. Participants in the familiar condition thought that the alignable attributes of brand A were more attractive than those of brand B [M_phone A_ = 5.09, M_phone A_ = 3.21; *t*(59) = 10.17, *p* < 0.001], and the nonalignable attributes of brand B were more attractive than those of brand A [M_phone A_ = 4.50, M_phone A_ = 5.48; *t*(59) = −5.22, *p* < 0.001]. In addition, the participants in the unfamiliar condition also thought that the alignable attributes of brand A were more attractive than those of brand B [M_monitoring player A_ = 5.04, M_monitoring player B_ = 2.96; *t*(71) = 14.64, *p* < 0.001] and the nonalignable attributes of brand B were more attractive than those of brand A [M_monitoring player A_ = 4.14, M_monitoring player B_ = 5.07; *t*(59) = −5.10, *p* < 0.001].

##### Hypothesis Testing

To test our predictions, we conducted a mixed-design ANOVA on the purchase intention and brand evaluation. As for purchase intention, the main effect of brand [*F*(1,130) = 1.47, *p* > 0.1] and product familiarity [*F*(1,130) = 0.03, *p* > 0.1] were not significant, but most importantly, consistent with our hypotheses, we found a significant two-way interaction between brand and product familiarity [*F*(1,130) = 21.60, *p* < 0.01]. As for brand evaluation, the main effects of brand [*F*(1,130) = 3.64, *p* > 0.1] and product familiarity [*F*(1,130) = 1.00, *p* > 0.1] were not significant. Furthermore, consistent with our hypotheses, we found a significant two-way interaction between brand and product familiarity [*F*(1,130) = 35.33, *p* < 0.01].

**Figure 2:**
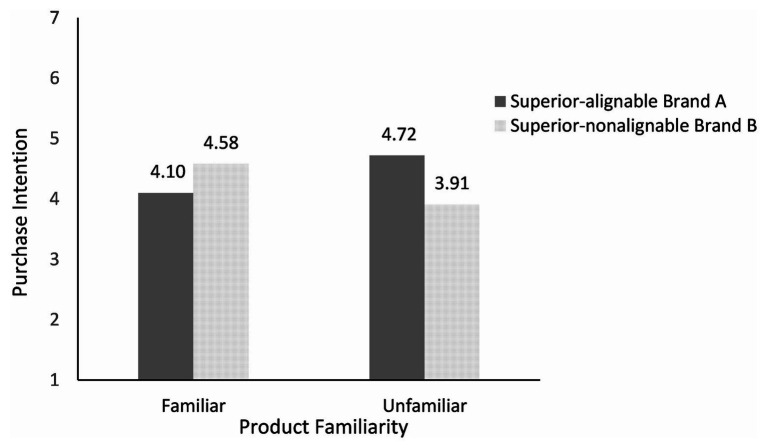
Effects of product familiarity and attribute alignability on purchase intention.

**Figure 3:**
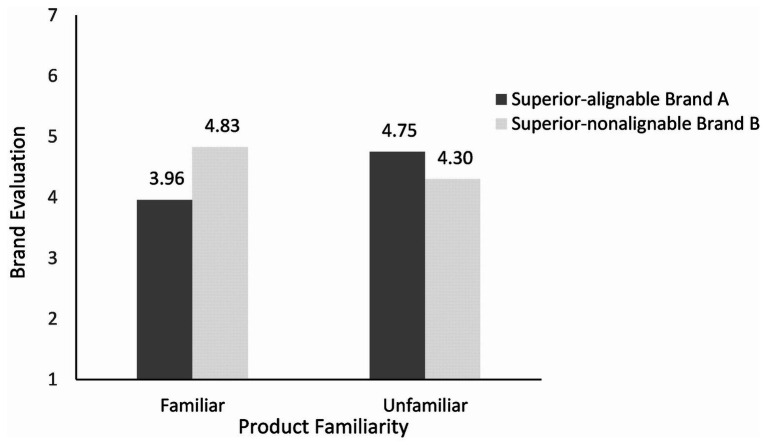
Effects of product familiarity and attribute alignability on brand evaluation.

To explore these interactions further, we conducted the paired-samples *t* test. As shown in Figures 2, 3, the results showed that in the familiar condition, the participants had a higher purchase intention for brand B (superior in nonalignable attributes) than brand A [superior in alignable attributes; M_A_ = 4.10, M_B_ = 4.58; *t*(59) = −2.09, *p* < 0.05]. The evaluation of brand B is also higher than that of brand A [M_A_ = 3.96, M_B_ = 4.83; *t*(59) = −4.36, *p* < 0.01]. In the unfamiliar condition, the participants had a higher purchase intention for brand A than brand B [M_A_ = 4.72, M_B_ = 3.91; *t*(71) = 4.85, *p* < 0.01]. The evaluation of brand A is also higher than that of brand B [M_A_ = 4.75, M_B_ = 4.30; *t*(71) = 3.89, *p* < 0.01]. These results supported Hypothesis 1.

##### Mediation Analysis

Referring to the mediation test method used in the research of Sun et al. (2019), we created a relative purchase intention index by dividing the purchase intention score of brand A by the purchase intention score of brand B. The larger the index is, the stronger the participants’ purchase intention for the superior-alignable brand A over the superior-nonalignable brand B is. We also calculated a relative perceived diagnosticity index by dividing the perceived diagnosticity score of brand A by the perceived diagnosticity score of brand B. Product familiarity is coded as a dummy variable (1 = familiar condition, 0 = unfamiliar condition). A mediation analysis using four regressions (Baron and Kenny, 1986) was performed. The results show that product familiarity (the independent variable) is a significant predictor of relative purchase intention (the dependent variable; β = 0.33, *p* < 0.01), and product familiarity is also a significant predictor of relative perceived diagnosticity (the mediator; β = 1.26, *p* < 0.01). In addition, relative perceived diagnosticity is a significant predictor of relative purchase intention (β = 0.25, *p* < 0.01). However, when product familiarity and relative perceived diagnosticity are included in the regression model for relative purchase intention, only relative perceived diagnosticity remains significant (β = 0.22, *p* = 0.05), and product familiarity is not significant (β = 0.06, *p* > 0.1). This has thereby provided support for H2, which states that the interaction effect of attribute alignability and product familiarity on product purchase is fully mediated by perceived diagnosticity.

##### Discussion

Study 1 mainly examined the influence of product familiarity on the attribute alignability dependency. The experimental outcome supports Hypotheses 1 and 2. When comparing products, consumers rely more on the nonalignable attributes if they have a high degree of product familiarity, while relying more on the alignable attributes if they have a low degree of product familiarity. With different levels of product familiarity, consumers’ dependence on attribute alignability in purchase decision-making is driven by perceived diagnosticity.

### Study 2

Study 2 further explored the impact of consumers’ personal characteristics on the attribute alignability dependency. Specifically, we investigated the interaction effect of product familiarity and self-construal on the dependence on attribute alignability.

#### Participants and Design

Two hundred students (44.4% females, aged from 18 to 25 years) participated in this study. Using the same stimulus and fictional brands as in study 1, study 2 employed a 2 (brand: brand A with superior alignable attributes vs. brand B with superior nonalignable attributes) × 2 (product familiarity: familiar vs. unfamiliar) × 2 (self-construal: independent vs. interdependent) mixed design, with the first factor being a within-subjects variable, the second factor being a between-subjects variable, and the third factor being a measured variable. The same as study 1, three blocks of attributes of both brands were assigned through Latin square design, in order to eliminate the influence of individual differences. Participants were randomly assigned to either the familiar or unfamiliar condition. The return of questionnaires with valid responses was 90%.

##### Procedure

This experiment was mostly similar to study 1. We asked participants to finish the Self-construal Scale (SCS; Singelis, 1994) at the beginning of the experiment. The SCS has 12 items for independence and 12 items for interdependence, scored by the Likert’s seven-point scale (1 = “totally inconsistent with me”; 7 = “totally consistent with me”). Next, participants went through the same process as in study 1 and evaluated the attractiveness of each attribute and purchase intentions of two brands. The entire experiment process was completed in approximately 40 min.

#### Results

##### Manipulation Check

The manipulation check confirmed the effectiveness of all our manipulation. The results revealed that participants in the familiar condition were indeed more familiar with the product than those in unfamiliar condition [M_phone_ = 5.99, M_monitoring player_ = 2.10; *t*(178) = 21.66, *p* < 0.001]. Moreover, the participants in the familiar condition thought that the alignable attributes of brand A were more attractive than those of brand B [M_phone A_ = 5.15, M_phone B_ = 3.31; *t*(93) = 12.06, *p* < 0.001], while the nonalignable attributes of brand B were more attractive than those of brand A [M_phone A_ = 4.97, M_phone B_ = 5.56; *t*(93) = −4.72, *p* < 0.001]. In addition, the participants in the unfamiliar condition thought that the alignable attributes of brand A were more attractive than those of brand B [M_monitoring player A_ = 5.09, M_monitoring player B_ = 2.92; *t*(85) = 17.10, *p* < 0.001], while the nonalignable attributes of brand B were more attractive than those of brand A [M_monitoring player A_ = 4.14,M_monitoring player B_ = 5.08; *t*(85) = −5.58, *p* < 0.001].

##### Hypothesis Testing

To test our predictions, we conducted a random design ANOVA analysis. Consistent with our hypotheses, we found a significant two-way interaction between brand and product familiarity [*F*(1,358) = 19.147, *p* < 0.001] and a significant three-way interaction among brand, product familiarity, and self-construal [*F*(1,358) = 5.432, *p* < 0.05].

**Figure 4:**
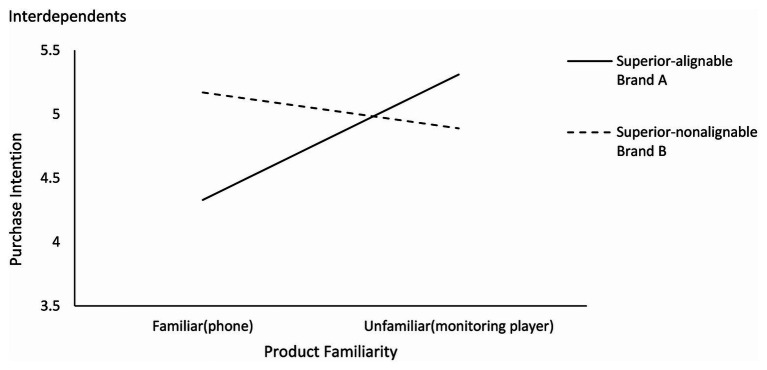
Effects of product familiarity and attribute alignability on purchase intention for interdependents (study 2).

**Figure 5:**
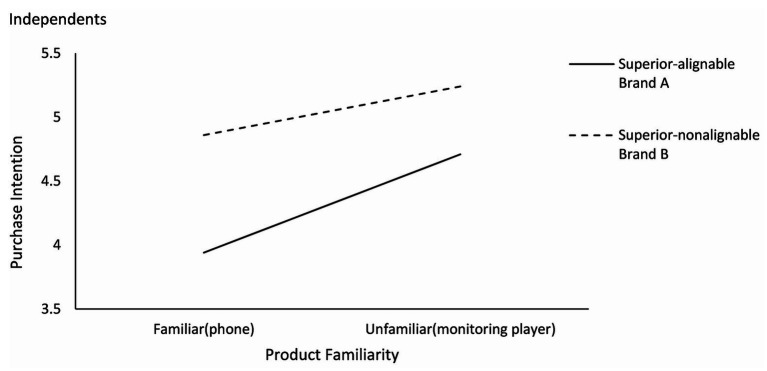
Effects of product familiarity and attribute alignability on purchase intention for independents (study 2).

Specifically, as shown in Figures 4, 5, the interdependents had a higher purchase intention for brand B (superior in nonalignable attributes) than brand A [superior in alignable attributes; M_A_ = 4.33, M_B_ = 5.17; *F*(1,102) = 24.035, *p* < 0.001] in the familiar condition and a higher purchase intention for brand A than brand B [M_A_ = 5.31, M_B_ = 4.89; *F*(1,92) = 4.561, *p* < 0.05] in the unfamiliar condition. Independents had a higher purchase intention for brand B than brand A in both familiar condition [M_A_ = 3.94, M_B_ = 4.86; *F*(1,82) = 21.493, *p* < 0.001] and unfamiliar condition [M_A_ = 4.71, M_B_ = 5.24; *F*(1,76) = 8.758, *p* < 0.01].

The results supported Hypothesis 3.

##### Discussion

The results of study 2 not only demonstrated Hypothesis 1 again but also provided support for Hypothesis 3. The results showed that the interdependents rely more on alignable attributes in purchase decision-making when unfamiliar with the product, while relying more on nonalignable attributes when familiar with the product. The independents, however, rely more on nonalignable attributes regardless of the degree of product familiarity.

### Study 3

In study 1 and study 2, the difference in product category may affect the results of the experiment. In order to eliminate this confounded impact and garner more confidence in our hypothesis, we manipulated product familiarity in one product category in study 3.

#### Pretest and Stimulus

In study 3, we chose digital camera as the stimulus. The attributes of digital camera are relatively complex, and most people only have a simple understanding on digital camera, but are not very familiar with it (Moreau et al., 2001). The same as study 1, we used two fictional brands for digital camera to eliminate the influence of brand preference. We also conducted two pretests as in study 1 to ensure the validity of the experimental materials. Fifty-five students participated in the first pretest and screened out the nine most important attributes (effective pixels, shutter speed, battery, support for external power, optical zoom, manual mode, anti-shake function, aperture range, and sensor size). We randomly divided them into three blocks of three attributes each. Then, we randomly recruited 30 students to rate the importance of three blocks in the second pretest. The importance ratings showed no significant difference across the three blocks of digital camera attributes [M_1_ = 6.12, M_2_ = 5.84 vs. M_3_ = 5.82; *F*(2,87) = 1.81, *p* > 0.01].

#### Participants and Design

Three hundred and eighty students (50.1% females, aged from 18 to 25 years) participated in this experiment. Study 3 employed a 2 (brand: brand A with superior alignable attributes vs. brand B with superior nonalignable attributes) × 2 (product familiarity: familiar vs. unfamiliar) × 2 (self-construal: independent vs. interdependent) mixed design, with the first factor as a within-subjects variable, the second factor as a between-subjects variable, and the third factor as a measured variable. The same as study 1, three blocks of attributes of both brands were assigned by Latin square design. Participants were randomly assigned to either the familiar or unfamiliar condition. The return of questionnaires with valid responses was 94.5%.

##### Procedure

At the beginning of the experiment, in order to manipulate product familiarity, we described each attribute and function of digital camera in detail to the participants in the familiar condition, while describing nothing in the unfamiliar condition. Next, participants went through the same process as in study 2. The entire experiment process was completed in approximately 40 min.

#### Results

##### Manipulation Check

The manipulation check confirmed the effectiveness of all our manipulation. The results revealed that participants in the familiar condition were indeed more familiar with the digital camera than those in the unfamiliar condition [M_familiar_ = 6.48, M_unfamiliar_ = 3.15; *t*(357) = 25.26, *p* < 0.001]. Moreover, participants in the familiar condition thought that the alignable attributes of brand A were more attractive than those of brand B [M_A_ = 5.81, M_B_ = 3.88; *t*(131) = 11.48, *p* < 0.001], while the nonalignable attributes of brand B were more attractive than those of brand A [M_A_ = 4.34, M_B_ = 5.68; *t*(131) = −9.654, *p* < 0.001]. In addition, the participants in the unfamiliar condition also thought that the alignable attributes of brand A were more attractive than those of brand B [M_A_ = 5.44, M_B_ = 4.41; *t*(226) = 8.492, *p* < 0.001], while the nonalignable attributes of brand B were more attractive than those of brand A [M_A_ = 3.72, M_B_ = 5.58; *t*(226) = −15.05, *p* < 0.001].

##### Hypothesis Testing

We conducted a random design ANOVA analysis. Consistent with our hypotheses, we found a significant two-way interaction between brand and product familiarity [*F*(1,716) = 35.597, *p* < 0.01] and a significant three-way interaction between brand, product familiarity, and self-construal [*F*(1,716) = 38.748, *p* < 0.01].

**Figure 6:**
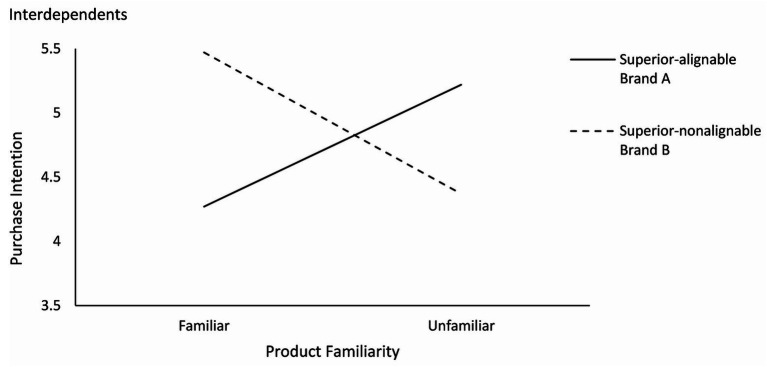
Effects of product familiarity and attribute alignability on purchase intention for interdependents (study 3).

**Figure 7:**
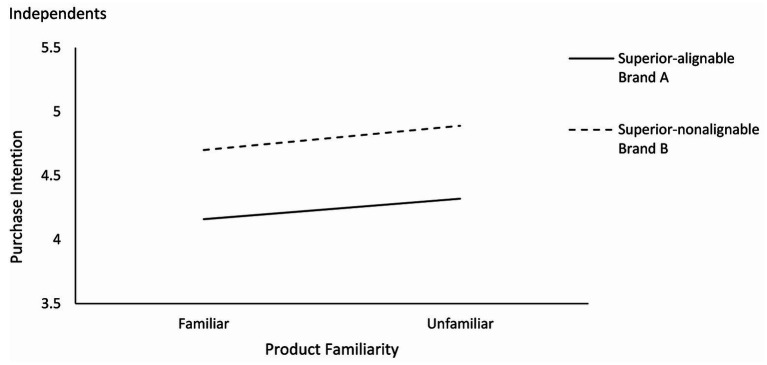
Effects of product familiarity and attribute alignability on purchase intention for independents (study 3).

Specifically, as shown in Figures 6, 7, the interdependents had a higher purchase intention for brand B (superior in nonalignable attributes) than brand A [superior in alignable attributes; M_A_ = 4.27, M_B_ = 5.47; *F*(1,142) = 11.031, *p* < 0.01] in the familiar condition, and a higher purchase intention for brand A than brand B [M_A_ = 5.22, M_B_ = 4.37; *F*(1,290) = 50.935, *p* < 0.001] in the unfamiliar condition. Independents had a higher purchase intention for brand B than brand A in both familiar condition [M_A_ = 4.16, M_B_ = 4.70; *F*(1,82) = 34.660, *p* < 0.001] and unfamiliar condition [M_A_ = 4.32, M_B_ = 4.89; *F*(1,160) = 15.418, *p* < 0.001].

##### Discussion

Study 3 manipulated product familiarity in one product category. It not only eliminated the potential impact caused by product category differences in study 2 but also validated the conclusions of study 2 again in a new product category. In general, the results further lend credence to our hypothesis that consumers’ dependency on attribute alignability in purchase decision-making varies due to their familiarity with the product and their personal characteristics. Specifically, the interdependents rely more on alignable attributes when unfamiliar with the product and rely more on nonalignable attributes when familiar with the product. The independents, however, rely more on nonalignable attributes regardless of the degree of product familiarity.

## General Discussion

Previous studies on the Structural Alignment Model suggest that consumers rely more on alignable than nonalignable attributes when comparing alternatives in most cases. We suppose that consumers’ product familiarity and self-construal play significant roles in regulating consumers’ reliance on attribute alignability. Across three studies, we confirmed the following: (1) Consumers’ familiarity with product will influence their reliance on attribute alignability when making purchase decisions. Consumers familiar with the product rely more on nonalignable attributes, while consumers unfamiliar with the product rely more on alignable attributes. (2) Perceived diagnosticity plays a mediating role in this process. Consumers familiar with the product are more likely to consider nonalignable attributes to be diagnosable, whereas consumers unfamiliar with the product consider alignable attributes as diagnostic information. Specifically, consumers need to evaluate product attributes from their prior consumption experience and other clues when making decisions. The less experience the consumers have, the less likely they are to believe their own inferences, meaning that they turn to rely more on the information clearly provided. Compared with nonalignable attributes, alignable attributes are clearer and easier to deal with, which makes them more diagnostic to low-familiarity consumers. While high-familiarity consumers have enough experience to process complex nonalignable attributes information, thus considering nonalignable attributes to be more differentiated and diagnostic. Therefore, they will be more likely to rely on nonalignable attributes in decision-making (3). The dependency of consumers with different product familiarity on attribute alignability will be further influenced by consumers’ personal characteristics and self-construal. The interdependents will rely more on alignable attributes when unfamiliar with the product and rely more on nonalignable attributes when familiar with the product. The independents, however, will rely more on nonalignable attributes regardless of the degree of product familiarity. For independents, even if they are unfamiliar with products, they tend to take a positive attitude and dare to take risk, which makes them believe their inferences and rely more on unique nonalignable attributes.

### Theoretical Contributions

This research offers two theoretical contributions. Firstly, this work contributes to the research on Structural Alignment Model. The comparison between products is involved in most purchase decision-making processes, where a product can be considered as a mixture of alignable and nonalignable attributes. Therefore, it is important to further explore consumer’s reliance on attribute alignability. Although prior researches have explored the influence of personal or situational factors on the attribute alignability, lack of in-depth discussion from the perspective of consumer cognition still stands. This research introduces and validates the moderating effect of product familiarity and self-construal on consumers’ dependency on attribute alignability, obtaining a more specific model of the influence of attribute alignability on purchasing decision, enriching the theory of attribute alignability and Structural Alignment Model. Moreover, we also explain the mechanism of this process, in which consumers with different product familiarity perceive significant differences in diagnosticity of alignable and nonalignable attributes. We clarify the cognitive process behind consumers’ decision-making behavior and further reveal the consumer black box of reliance on attribute alignability in purchase decision-making process.

Secondly, self-construal was proposed and defined as how individuals view themselves in relation to the social circumstances (Markus and Kitayama, 1991). It is an individual trait and influenced by people’s long-term growth environment. It has a great influence on consumers’ mindset and behavior patterns. We examine that the chronic self-construal and product familiarity have an interactive effect on consumers’ dependency on attribute alignability. Our study provides effective supplements to the existing self-construal literature and consumer decision-making literature through demonstrating the important role of individual differences and long-term cultural differences in the consumer decision-making process.

### Practical Implications

This research provides important practical implications to corporate managers, assisting them to formulate relevant marketing strategies in accordance with individual characteristics of consumers in terms of product function design, positioning and marketing communication strategies.

Firstly, managers can design product attributes, establish brand position, and adopt differentiated communication strategies based on the characteristics of target consumers. If the products or services provided by the company are mainly aimed at the independents or consumers with rich product experience, managers should highlight the nonalignable attributes of the product in product design and positioning, and emphasize the unique function of the product that are different from competitors. If the target consumers are the consumers with interdependent self and unfamiliar with the product, the managers ought to use the communication strategy that can stimulate consumers to compare with other brands in the alignable attributes. In addition, managers also need to emphasize the utility of relevant attributes to better activate consumers’ diagnostic judgment of product attributes, so as to enhance consumers’ willingness to buy.

Secondly, managers should also consider the impact of product attribute alignability on consumer decision-making in the product life cycle management. Generally speaking, in the early stages of the product life cycle, consumers are relatively less familiar with the product, so managers should highlight alignable attributes. In the maturity stage, managers can emphasize nonalignable attributes and attract consumers by differentiated and innovative attributes.

### Limitations and Future Research

This study sheds light on the interaction of product familiarity and self-construal on attribute alignability dependency when making purchase decisions. However, there are still several limitations, which can be further explored in future research.

Firstly, this research only validates the moderating effect of consumers’ chronic self-construal in the Chinese context. However, in the actual purchase process, consumers are often affected by contextual clues. Therefore, in future research, we can further study the influence of situational self-construal and explore how to stimulate situational self-construal through contextual cues. In this way, marketers can draw consumers’ attention to alignable or nonalignable attributes and better display the superior attributes of their companies’ products. Moreover, it is expected to validate the moderating effect of consumers’ individual trait among consumers from different countries and cultural backgrounds, which is meaningful for multinational companies.

In addition, this research only explores one of the boundary conditions that affect consumers’ dependency on attribute alignability, that is, the interaction between product familiarity and self-construal. There are still many other valuable segmentation variables whose effects deserve further exploration.

Moreover, this study has explored the moderating role of consumers’ individual factors in the influence of attribute alignability on brand evaluation and product purchase. In future research, we can further explore more interaction effects among consumers’ personal factors and attribute alignability on perceived quality, intention to recommend, impulsive consumption, pay premium green consumption, etc.

## Data Availability Statement

The raw data supporting the conclusions of this article will be made available by the authors, without undue reservation.

## Ethics Statement

The studies involving human participants were reviewed and approved by the School of Management, Jinan University, China. The patients/participants provided their written informed consent to participate in this study.

## Author Contributions

YZ, YW, and MH jointly designed, analyzed, wrote this paper, undertook all testing, and data collection. All authors contributed to the article and approved the submitted version.

### Conflict of Interest

The authors declare that the research was conducted in the absence of any commercial or financial relationships that could be construed as a potential conflict of interest.

## References

[ref1] Aaker J. Schmitt B. (2001). Culture-dependent assimilation and differentiation of the self - Preferences for consumption symbols in the United States and China. J. Cross-Cult. Psychol. 32, 561–576. 10.1177/0022022101032005003

[ref2] Alba J. W. Hutchinson J. W. (1987). Dimensions of consumer expertise. J. Consum. Res. 13, 411–454. 10.1086/209080

[ref500] Baron R. M. Kenny D. A. (1986). The moderator–mediator variable distinction in social psychological research: conceptual, strategic, and statistical considerations. J. Pers. Soc. Psychol. 51, 1173–1182. 10.1037/0022-3514.51.6.1173 3806354

[ref3] Carpenter G. S. Glazer R. Nakamoto K. (1994). Meaningful brands from meaningless differentiation: the dependence on irrelevant attributes. J. Mark. Res. 31, 339–350. 10.1177/002224379403100302

[ref4] Coupey E. Irwin J. R. Payne J. W. (1998). Product category familiarity and preference construction. J. Consum. Res. 24, 459–468. 10.1086/209521

[ref5] Escalas J. E. Bettman J. R. (2005). Self-construal, reference groups, and brand meaning. J. Consum. Res. 32, 378–389. 10.1086/497549

[ref6] Feldman J. M. Lynch J. G. (1988). Self-generated validity and other effects of measurement on belief, attitude, intention, and behavior. J. Appl. Psychol. 73, 421–435. 10.1037/0021-9010.73.3.421

[ref8] Gentner D. Markman A. B. (1994). Structural alignment in comparison: no difference without similarity. Psychol. Sci. 5, 152–158. 10.1111/j.1467-9280.1994.tb00652.x

[ref7] Gentner D. Markman A. B. (1997). Structure mapping in analogy and similarity. Am. Psychol. 52, 45–56. 10.1037/0003-066X.52.1.45

[ref9] Gursoy D. Chi C. G. (2008). “Travelers’ information search behavior” in Handbook of Hospitality Marketing Management. eds. O. Haemoon and P. Abraham (Abingdon: Routledge).

[ref10] Hamilton R. W. Biehal G. J. (2005). Achieving your goals or protecting their future? The effects of self-view on goals and choices. J. Consum. Res. 32, 277–283. 10.1086/432237

[ref11] Han S.-P. Shavitt S. (1994). Persuasion and culture: advertising appeals in individualistic and collectivistic societies. J. Exp. Soc. Psychol. 30, 326–350. 10.1006/jesp.1994.1016

[ref12] Heath C. Tversky A. (1991). Preference and belief: ambiguity and competence in choice under uncertainty. J. Risk Uncertain. 4, 5–28. 10.1007/BF00057884

[ref13] Heine S. J. Lehman D. R. Markus H. R. Kitayama S. (1999). Is there a universal need for positive self-regard? Psychol. Rev. 106, 766–794. 10.1037/0033-295X.106.4.766, PMID: 10560328

[ref14] Hsee C. K. (1996). The evaluability hypothesis: An explanation for preference reversals between joint and separate evaluations of alternatives. Organ. Behav. Hum. Decis. Process. 67, 247–257. 10.1006/obhd.1996.0077

[ref15] Jain S. P. Desai K. K. Mao H. (2007). The influence of chronic and Situational self-construal on categorization. J. Consum. Res. 34, 66–76. 10.1086/513047

[ref16] Kardes F. R. Kalyanaram G. (1992). Order-of-entry effects on consumer memory and judgment: An information integration perspective. J. Mark. Res. 29, 343–357. 10.1177/002224379202900305

[ref17] Kerstetter D. Cho M. H. (2004). Prior knowledge, credibility and information search. Ann. Tour. Res. 31, 961–985. 10.1016/j.annals.2004.04.002

[ref18] Lee A. Y. Aaker J. L. Gardner W. L. (2000). The pleasures and pains of distinct self-construals: the role of interdependence in regulatory focus. J. Pers. Soc. Psychol. 78, 1122–1134. 10.1037/0022-3514.78.6.1122, PMID: 10870913

[ref19] Lee J. A. Kacen J. J. (2008). Cultural influences on consumer satisfaction with impulse and planned purchase decisions. J. Bus. Res. 61, 265–272. 10.1016/j.jbusres.2007.06.006

[ref20] Lee B. K. Lee W. N. (2016). The effect of structural alignment on choice-process satisfaction and preference formation: The moderating role of self-construal. J. Bus. Res. 69, 2747–2755. 10.1016/j.jbusres.2015.11.010

[ref21] Lynch J. G. Jr. Marmorstein H. Weigold M. F. (1988). Choices from sets including remembered brands: use of recalled attributes and prior overall evaluations. J. Consum. Res. 15, 169–184. 10.1086/209155

[ref22] Ma Z. F. Yang Z. Y. Mourali M. (2014). Consumer adoption of new products: independent versus interdependent self-perspectives. J. Mark. 78, 101–117. 10.1509/jm.12.0051

[ref23] Malkoc S. A. Zauberman G. Ulu C. (2005). Consuming now or later? The interactive effect of timing and attribute alignability. Psychol. Sci. 16, 411–417. 10.1111/j.0956-7976.2005.01549.x, PMID: 15869702

[ref24] Markman A. B. Gentner D. (1993). Structural alignment during similarity comparisons. Cogn. Psychol. 25, 431–467. 10.1006/cogp.1993.1011

[ref25] Markman A. B. Gentner D. (1997). The effects of alignability on memory. Psychol. Sci. 8, 363–367. 10.1111/j.1467-9280.1997.tb00426.x

[ref26] Markus H. R. Kitayama S. (1991). “Cultural variation in the self-concept” in The self: Interdisciplinary approaches. eds. Strauss J. Goethals G. R. (New York, NY: Springer).

[ref28] Medin D. L. Goldstone R. L. Markman A. B. (1995). Comparison and choice: Relations between similarity processes and decision processes. Psychon. Bull. Rev. 2, 1–19. 10.3758/BF03214410, PMID: 24203588

[ref29] Moreau C. P. Markman A. B. Lehmann D. R. (2001). “What is it?” Categorization flexibility and consumers’ responses to really new products. J. Consum. Res. 27, 489–498. 10.1086/319623

[ref30] Murshed F. Nagpal A. Moosa A. (2018). Self-customisation and attribute alignability: role of utilitarian versus hedonic consumption. J. Res. Consum. 83–115.

[ref31] Nam M. Wang J. Lee A. Y. (2012). The difference between differences: how expertise affects diagnosticity of attribute alignability. J. Consum. Res. 39, 736–750. 10.1086/664987

[ref32] Pham M. T. Avnet T. (2004). Ideals and oughts and the reliance on affect versus substance in persuasion. J. Consum. Res. 30, 503–518. 10.1086/380285

[ref34] Rogers M. D. (2003). Risk analysis under uncertainty, the precautionary principle, and the new EU chemicals strategy. Regul. Toxicol. Pharmacol. 37, 370–381. 10.1016/S0273-2300(03)00030-8, PMID: 12758217

[ref35] Singelis T. M. (1994). The measurement of independent and interdependent self-construals. Personal. Soc. Psychol. Bull. 20, 580–591. 10.1177/0146167294205014

[ref36] Sun J. (2011). Consumer brand preference construction: the moderating role of evaluation mode. Manag. Rev. 12, 103–111. 10.14120/j.cnki.cn11-5057/f.2011.08.020

[ref37] Sun J. Keh H. T. Lee A. Y. (2012). The effect of attribute alignability on service evaluation: the moderating role of uncertainty. J. Consum. Res. 39, 831–847. 10.1086/665983

[ref38] Sun J. Keh H. T. Lee A. Y. (2019). Shaping consumer preference using alignable attributes: The roles of regulatory orientation and construal level. Int. J. Res. Mark. 36, 151–168. 10.1016/j.ijresmar.2018.12.001

[ref39] Sweeney J. C. Soutar G. N. Johnson L. W. (1999). The role of perceived risk in the quality-value relationship: A study in a retail environment. J. Retail. 75, 77–105. 10.1016/S0022-4359(99)80005-0

[ref40] Taylor-West P. Saker J. Champion D. (2020). Market segmentation strategies for complex automotive products. J. Strateg. Mark. 28, 266–283. 10.1080/0965254X.2018.1555548

[ref41] Tversky A. (1977). Features of similarity. Psychol. Rev. 84, 327–352. 10.1037/0033-295X.84.4.327

[ref42] Zhang S. Fitzsimons G. J. (1999). Choice-process satisfaction: the influence of attribute alignability and option limitation. Organ. Behav. Hum. Decis. Process. 77, 192–214. 10.1006/obhd.1999.2821, PMID: 10080913

[ref43] Zhang S. Kardes F. R. Cronley M. L. (2002). Comparative advertising: effects of structural alignability on target brand evaluations. J. Consum. Psychol. 12, 303–311. 10.1207/15327660260382342

[ref44] Zhang S. Markman A. B. (1998). Overcoming the early entrant advantage: the role of alignable and nonalignable differences. J. Mark. Res. 35, 413–426. 10.1177/002224379803500401

[ref45] Zhang S. Markman A. B. (2001). Processing product unique features: alignability and involvement in preference construction. J. Consum. Psychol. 11, 13–27. 10.1207/S15327663JCP1101_2

[ref47] Zhou K. Z. Nakamoto K. (2007). How do enhanced and unique features affect new product preference? The moderating role of product familiarity. J. Acad. Mark. Sci. 35, 53–62. 10.1007/s11747-006-0011-3

